# A Glance at the Molecules That Regulate Oligodendrocyte Myelination

**DOI:** 10.3390/cimb44050149

**Published:** 2022-05-15

**Authors:** Shunqi Wang, Yingxing Wang, Suqi Zou

**Affiliations:** 1 Institute of Life Science & School of Life Sciences, Nanchang University, Nanchang 330031, China; wsqi@ncu.edu.cn (S.W.); 13017654792@163.com (Y.W.); 2 School of Basic Medical Sciences, Nanchang University, Nanchang 330031, China

**Keywords:** oligodendrocyte, myelination, proliferation, differentiation, maturation, hypomyelination, hypermyelination

## Abstract

Oligodendrocyte (OL) myelination is a critical process for the neuronal axon function in the central nervous system. After demyelination occurs because of pathophysiology, remyelination makes repairs similar to myelination. Proliferation and differentiation are the two main stages in OL myelination, and most factors commonly play converse roles in these two stages, except for a few factors and signaling pathways, such as OLIG2 (Oligodendrocyte transcription factor 2). Moreover, some OL maturation gene mutations induce hypomyelination or hypermyelination without an obvious function in proliferation and differentiation. Herein, three types of factors regulating myelination are reviewed in sequence.

## 1. Introduction

OL myelination is critical to the vertebrate central nervous system (CNS) function. It supports not only the myelinating cell in the CNS but also provides metabolic and trophic support to the myelinated axon. The myelin sheath is essential insulation surrounding axons for conduction in the nervous system. Hypermyelination or hypomyelination interferes with saltatory nerve conduction, causing neurological disabilities [1,2,3].

**Figure 1:**
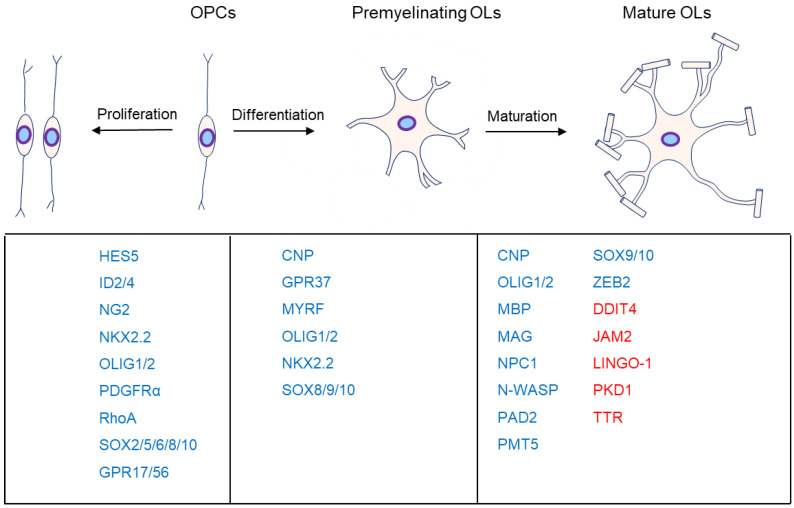
Major markers at the different stages of oligodendrocyte development. OL myelination is mainly divided into proliferation, differentiation, and maturation. The genes associated with the different stages are listed below them. Moreover, genes in blue are the positive markers in OPC proliferation, OL differentiation, or maturation. Specifically, genes in red are inhibitors in OL maturation.

OL progenitor cells (OPCs) come originally from neuroepithelial precursor cells and then proliferate and differentiate into premyelinating OLs, subsequently differentiating into myelinating OLs in the CNS (Figure 1), where an individual OL can myelinate up to 40 axonal segments [4,5,6,7]. Sequential generation of OPCs from specific germinal regions is reviewed by S. Mitew [8].

OPC proliferation and OL differentiation are the two main critical processes in myelination and remyelination. Despite some molecules having opposing functions in OPC proliferation and OL differentiation, the physiological processes they control are not opposed. Transitory expression of signaling molecules that drive OPC proliferation and block OL differentiation or maturation is required for early OPC content control within the CNS. Sequential transitory expression or modification of signaling molecules that drive OL differentiation or maturation and block OPC proliferation are required for OL lineage progression and proper myelination or remyelination. The molecules and the classic signaling pathway controlling the delicate and complicated balance among OPC proliferation, OL differentiation and maturation will be reviewed separately and sequentially.

## 2. OPC Proliferation

Myelination is a multi-step process from OPCs to OLs. Although OPC proliferation opposes OPC differentiation into OLs, OPC proliferation contributes to myelinating OLs. Therefore, in some respects, OPC proliferation is the first essential step for myelination and remyelination. Many factors have been found to stimulate this process, including classic signaling pathways and transcription factors such as WNT (Wingless-type MMTV integration site family), GPR56 (G protein-coupled receptor 56), GPR17, NG2 (chondroitin sulfate proteoglycan neuron-glia antigen 2), SOX2 (SRY (sex-determining region Y)-box transcription factor 2) and NOTCH1 (Notch receptor 1). Blocking the inhibitory factors is an attractive therapeutic approach to reforming myelin repair.

OPCs specifically express PDGFRα (platelet-derived growth factor receptor α-subunit) and NG2, downregulated during OL differentiation [9,10]. NG2 is regarded as a factor maintaining OPC proliferation and preventing OL differentiation. Cortical NG2-positive cells are highly dynamic, surveying the microenvironment with filopodia, extending growth cones, and continuously migrating. The NG2-positive cell maintains the unique territory via self-avoidance [11].

OPCs also express high HES5 (Hes family bHLH transcription factor 5), ID2 (inhibitor of DNA binding 2), ID4, SOX5, and SOX6, restricting differentiation and myelination. For example, ID2 and ID4 are downstream of BMP (bone morphogenetic protein) [12] and GPR17 [13]. *Id2* overexpression limits OPC differentiation, whereas *Id2* knockout accelerates OL differentiation [14]. The potential mechanism of ID2 and ID4 hindering differentiation may be the interaction with OLIG1 and OLIG2 to obstruct the latter’s activity [12,14].

### 2.1. Wnt Signaling Pathway

**Figure 2:**
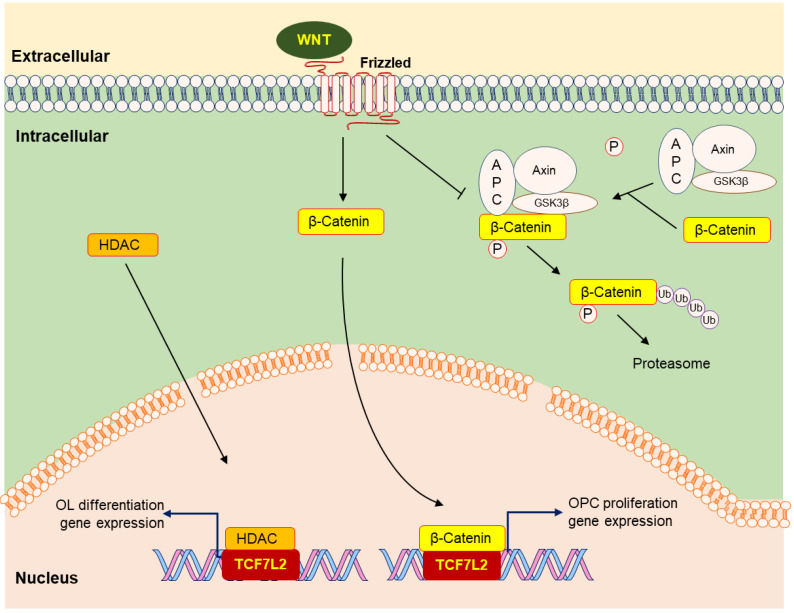
The role of the Wnt signaling pathway in OPC proliferation and OL differentiation. β-Catenin is the crucial effector in the canonical Wnt signaling pathway. When the WNT is absent, β-Catenin is phosphorated by GSK3β in the complex, and the phosphorylation is the signal for ubiquitination, leading to the digestion of β-Catenin. After WNT is activated, β-Catenin in the free state enters the nucleus and promotes OPC proliferation via binding with TCF7L2. HDAC may compete with β-Catenin to interact with TCF7L2 and switches TCF7L2 from an inhibitor to an activator in OL differentiation.

The Wnt signaling pathway (Wnt-β-Catenin-TCF7L2) promotes OL myelination with β-Catenin binding TCF7L2 (transcription factor 7-like 2); however, the TCF7L2 activation enhances OL differentiation when HDAC (histone deacetylase) replaces β-Catenin (Figure 2).

Canonical Wnt-β-Catenin signaling pathway (also termed Wnt-β-Catenin-TCF-LEF cascade) constrains OL differentiation to an immature state and strongly hampers differentiation [15,16,17,18]. Activating the canonical Wnt signaling pathway, the intranuclear factor TCF7L2 (also known as TCF4) serves as an essential negative regulator of OL differentiation during myelination and remyelination [17]. Under WNT3a treatment, differentiation of OL is strongly delayed or blocked [18], recruiting TCF7L2 to β-Catenin target genes to foster proliferation [17,19]. ID2 and ID4 are the potential targets of the transcriptional complex formed by β-Catenin and TCF7L2 in OL development. Moreover, a Wnt antagonist (rmFz-8/Fc) increases the number of immature OLs [18]. Strikingly, Hao Huang et al. found that *Id2* single mutation and *Id2/Id4* double mutations display a mild and transient precocity of OL differentiation [20]. *Id4* disruption has little effect on OL differentiation and maturation. Although *Id2*, but not *Id4*, is weakly expressed in OPCs, overexpression of *Id2* and *Id4* in embryonic chicken spinal cords strongly inhibits OL differentiation, suggesting *Id2/4* may not be the major repressors in OL differentiation [20].

Not surprisingly, β-Catenin disruption leads to enhancing the premyelinating OL. However, OL differentiation is not enhanced but reduced in *Tcf7l2* knockout mice [16,17], and differentiation is hampered in β-Catenin-inactivated mice [21], indicating the complexity of β-Catenin-TCF7L2. The potential mechanism is TCF7L2 interacting with HDAC [16]. Four classes of HDACs are recruited to form multiprotein transcriptional complexes and lead to impeding target gene expression. HDAC1 and HDAC2 are essential to OL differentiation. *Hdac1/2* double knockout in OLs leads to β-Catenin stabilization and translocation to nuclear, negatively mediating OL development by arresting Olig2 expression [16]. TCF7L2 is verified as a bipartite co-effector in β-Catenin, constraining OL differentiation. TCF7L2 dominant repressive form expression reinforces OL specification [16,19]. Hence, HDAC1/2 may compete with β-Catenin to interact with TCF7L2 controlling OL differentiation, and HDAC binding switches TCF7L2 from an inhibitor to an activator of OL differentiation [16,19]. Moreover, administration of 5-FU (5-Fluorouracil) results in fast demyelination in the CNS by disassociating the interaction between TCF7L2 and HDAC1/2 [22]. TCF7L2 acts like a molecular switch, inhibiting or promoting OL differentiation by associating with the different binding partners [8].

### 2.2. FGF2/FGFR1

**Figure 3:**
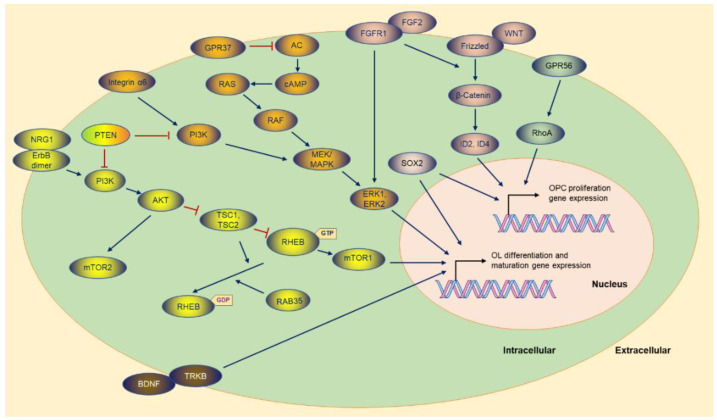
The molecules and the potential signaling pathway regulate the OPC proliferation and OL differentiation gene expression. Most of the molecules are exhibited, and the majority of the upstream molecules originate from the cytoplasmic membrane. The sign of blue arrows represents promoting, and the sign of red lines represents inhibiting. AC: Adenylate cyclase.

In the early stages, FGF2 (fibroblast growth factor 2) is known as a mitotic and neuroprotective factor, and its receptor is FGFR1 (fibroblast growth factor receptor 1) [23] (Figure 3). Maintaining the FGF2 level by administration stimulates OPC proliferation, hindering OL differentiation and maturation [23,24]. However, transient exposure to FGF2 benefits maturation [24,25]. In addition, FGF2 and FGFR1 are augmented in demyelinated lesions [26,27], and in *Fgf2* null mice, OL differentiation is elevated during remyelination [28]. Furthermore, *Fgf2*-deficient or *Fgfr1* knockdown accelerates oligodendrogenesis and remyelination, improving recovery after experimental demyelination [27,28,29]. The FGF signaling pathway contributes to the failure of remyelination.

FGF signaling is initially identified as an OPC proliferative signal retarding differentiation. However, in *Fgfr1/Fgfr2* double knockout mice, OPC proliferation and OL differentiation are not affected, and the onset of myelination is on time [30]. Rapid myelin growth in the CNS is firmly restrained, and the latent mechanism of hypomyelination in the double knockout mice is ERK1/2-MAPK (mitogen-activated protein kinase) activity being weakened [30]. The potential mechanism of FGF2 was recently found to hinder myelination, and it regulates activation of Wnt signaling via FGFR2 [31]. Inhibition of Wnt signaling is sufficient to abrogate the negative function of FGF2 in remyelination [31]. Additionally, fibrinogen hampers OL differentiation into myelinating OL via activating the BMP signaling pathway. Fibrinogen deficiency reforms the CNS remyelination [32].

### 2.3. GPR56

**Figure 4:**
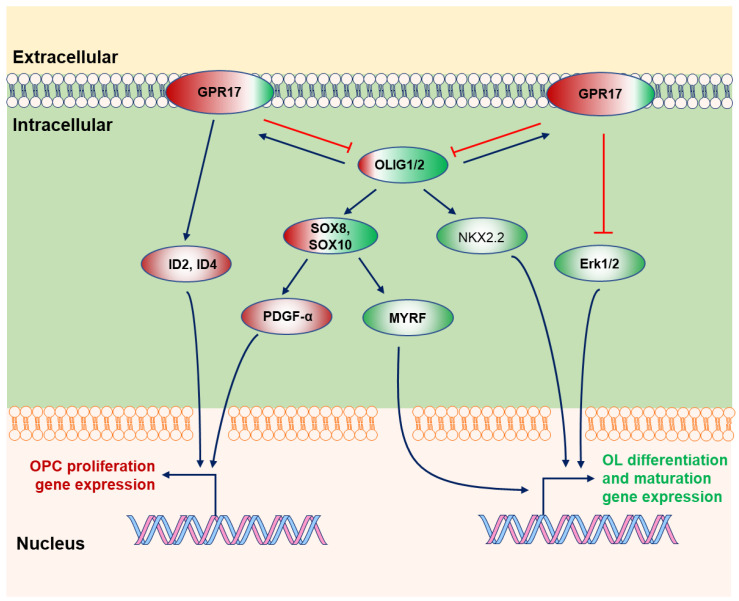
GPR17 promotes OPC proliferation and OLIG1/2 mainly induces OL differentiation. OLIG2 modulates *Gpr17* expression, and GPR17 acts as a negative feedback factor to *Olig1/2* expression. GPR17 mainly promotes OPC proliferation with inhibition in OL differentiation via ERK1/2. OLIG1/2 are the markers for OL family cells and have multiple roles in OL myelination via different downstream factors. Genes in the ellipse with different colors: the red color stands for the genes involved in OPC proliferation, and the green color refers to those correcting with OL differentiation and maturation. Different ratios of red and green in the ellipse imply the genes’ potential ratio in multiple processes. The sign of blue arrows represents promoting, and the sign of red lines represents inhibit.

In the CNS, the GPR family plays different roles, such as GPR56 modulating OPC proliferation (Figure 3) [33,34], GPR17 impeding OPC early differentiation [13] (Figure 4), and GPR37 restraining OL differentiation and maturation (Figure 3) [35]. GPR56, also known as ADGRG1 (adhesion G protein-coupled receptor G1), regulates many physiological processes via cell–cell and matrix communications. Loss function mutations of GPR56 lead to human brain malformation bilateral frontoparietal polymicrogyria (BFPP), including CNS hypomyelination [36,37]. GPR56 is required in OPC proliferation during developmental myelination in Zebrafish [34,38] and mice models [39] for enhancing RhoA (Ras homolog family member A) activity. GPR56 activates RhoA via coupling to Gα_12/13_ and maintains OPCs in an immature, proliferative state by preventing terminal differentiation [39]. Interestingly, *Gpr56* deficiency causes corrupted OPC proliferation and decreased myelinated axons. Further research has indicated that *Gpr56* conditioned knockout in microglia, astrocytes, or neurons does not impair developmental myelination [33]. Microglia-derived TG2 (transglutaminase-2) signals to GPR56 on OPCs depend on laminin, which was proposed to stimulate OPC proliferation [40]. *Tg2* conditioned knockout in microglia reduces OL numbers and myelination [40].

### 2.4. GPR17

During OL differentiation to premyelinating/myelinating OLs, positive and negative regulators coordinate the timing of OL maturation with complicated roles. GPR17 expression peak occurs in OPCs, and then its expression is gradually silenced in mature OLs [41,42,43]. GPR17 can be activated by either uracil nucleotides (UDP, UDP-glucose and UDP-galactose) or cysteinyl-leukotrienes (LTC4, LTD4, and LTE4) [41,44,45,46,47,48,49], leading to potent inhibition of intracellular cyclic adenosine monophosphate (cAMP) formation (Figure 4) [13,41,42,50] in OPCs. Inflammation-elicited factors may activate GPR17, preventing OPCs from differentiation and proliferating [51]. Therefore, GPR17 in OPCs is a cell-intrinsic timer of myelination [13,42,43,50,52]. Gpr17 promotes OPC proliferation and impedes OL differentiation and myelination via inducing ID2/4 [13]. OLIG1/2, a primary helix-loop-helix transcription factor, benefits OL maturation and remyelination. Although *OLIG1/2* regulates *Gpr17* expression, GPR17 acts as a negative feedback factor to OLIG1/2 [13]. *Gpr17* overexpression bridled OL differentiation and maturation in vitro and in vivo; conversely, OL myelination started early in *Gpr17*-deficient mice [13].

Although GPR17^+^/NG2^+^ glia prefer to remain undifferentiated from GPR17^−^/NG2^+^ cells, the double-positive cell effectively reacts to the damage and matures [53]. Chromatin immunoprecipitation (ChIP) sequencing indicated that OLIG2 and GPR17 are critical to regulating OL survival [54]. Following OL injury upregulating *Olig2* expression, OLIG2 transcriptionally activates *Gpr17* expression by targeting the *Gpr17* locus. Then, activated GPR17 stalls OL survival via enhancing *Xaf1* (XIAP-associated factor 1, A pro-apoptotic gene) expression and downregulating the PKA-cAMP-CREB signaling pathway [54,55]. Consistently, loss or block of GPR17 results in a rapid onset of remyelination by elevating ERK1/2 activity (phosphor-ERK1/2) [54,56].

J Wang et al. found that sustained microglial activation restrains OL myelination, and interruption of GPR17 reinforces OL differentiation [51]. Therefore, co-manipulating microglia and GPR17 can cause robust myelination of regenerated axons in the inflammatory environment after injury [51].

### 2.5. SOX2

Maintained *Sox2* expression restrains myelination and remyelination in mice [57] (Figure 3). Although *Sox2* expression is low in OPCs [58,59], early research proposed that SOX2 sustains OPC proliferation and restrains OL differentiation [59,60]. *Sox2* expression transiently boosts OL differentiation and regeneration during remyelination [61]. Strikingly, recent research demonstrated that SOX2 is essential for OPC proliferation and OL differentiation [61,62]. Unquestionably, *Sox2*-deficient mice display severe ataxia and tremors, a classical phenotype of hypomyelination [61].

### 2.6. NOTCH1

Notch signal constrains OPC differentiation because it maintains OPC in an undifferentiation state [63,64] (Figure 3), and *Notch1* haploinsufficiency leads to precocious myelination [65]. Notch limits OPC differentiation via transcription factor HES5, which competes with SOX10 to obstruct MBP (myelin basic protein) transcription [66]. The Notch pathway also is the potential target of bisphenol-A (BPA) and curcumin, and several genes are engaged in it, such as *Notch1*, *Hes1*, and *Mib1*(MIB E3 ubiquitin protein ligase 1). BPA impairs the myelination process and neurogenesis, resulting in cognitive dysfunctions. In contrast, curcumin reversed the impeding of BPA myelination by enhancing the Notch signaling pathway [67]. Thus, Notch signaling alteration may disrupt the proliferation of OPCs [67].

### 2.7. SHH

SHH (Sonic hedgehog) has been identified as a critical factor for OL lineage specification, and it controls both OPC proliferation and OL differentiation. *Shh* misexpression can induce ectopic OLs, and conversely, ablation of SHH signals leads to OL loss [18,68,69]. The SHH signaling pathway contributes to oligodendrogenesis during corpus callosum myelination in young mice or remyelination in adult mice [70,71,72,73]. SHH signaling inactivation leads to a dose-dependent MBP and MAG (myelin-associated glycoprotein) decrease in OLs [73]. *Shh*, *Smo* (smoothened, frizzled class receptor), and *Gli1* (GLI family zinc finger 1) are the essential components of the SHH signaling pathway, and BOC (BOC cell adhesion associated, oncogene regulated) is an SHH receptor. Mutant mice exhibit impeded myelination because of a decrease in OL density [69].

## 3. OL Differentiation

OLs generated from OPCs are fundamental to myelin formation, and all myelination processes are composed of OPC proliferation, OL differentiation, and maturation. Although a supra-threshold axon diameter (>0.3 μm) is necessary for axonal myelination during optic nerve development, OL differentiation is independent of dynamic signals from the myelinated neuron [74]. Supra-threshold axon diameter is only an insufficient necessary factor.

Notably, the first territories to be occupied by OPCs are not necessarily the first regions to be myelinated [75], suggesting a potential regulation of other environmental factors. However, dynamic neuronal signals such as transcriptional changes or neuronal activity may also mediate physiological processes, such as OL differentiation and maturation [76]. The optic nerves are almost myelinated, but most brain regions are partially myelinated, even though the axon caliber is far more than the supra-threshold. Consequently, potential inhibitory factors or repulsive signals likely prevent the onset of myelination [77]. Conversely, it is also possible that attractive factors or signals exist to initiate the onset of myelination [74].

### 3.1. AKT-mTOR

AKT (AKT serine/threonine kinase 1), as an essential effector of PI3K (phosphatidylinositol-4,5-bisphosphate 3-kinase), promotes myelination in the CNS [78,79] through the mTOR (mechanistic target of rapamycin) pathway (Figure 3). Akt stimulates axonal wrapping and raises myelin thickness by the mTOR pathway. Moreover, maintaining AKT activation induces hypermyelination or demyelination [80]. Accordingly, sustaining the PI3K signaling pathway in OPCs results in gradual hypermyelination, leading to leukodystrophy [81]. Conversely, PTEN (phosphatase and tensin homolog) [82], as an inhibitor of the PI3K-Akt-mTOR signaling pathway, negatively regulates myelination, and DLG1 (discs large MAGUK scaffold protein 1, an interactor of PTEN) has a similar effect during myelination [81,83]. CNP (2,3-cyclic nucleotide 3-phosphodiesterase) controlling constitutive AKT activation boosts MBP staining and increases the corpus callosum size, with pronounced hypermyelination in small-caliber axons [80].

mTOR, as the downstream of the PI3K-AKT signaling pathway, is the core element of two complexes (mTORC1 and mTORC2) with different functions in the complex [82,84]. Ablation of RAPTOR (regulatory-associated protein of MTOR complex 1), as an element of the mTORC1, leads to hypomyelination. Conversely, RICTOR (RPTOR independent companion of MTOR complex 2) deficiency does not affect myelination as an essential element of the mTORC2 [82].

Tuberous sclerosis protein 1 (TSC1) and TSC2 negatively regulate mTORC1 signaling [82,85,86], and downregulation of mTORC1 is essential for OPC differentiation and the subsequent myelination initiation. TSC2, as a member of GAP (GTPase-activating protein), constrains mTORC1 by stimulating the GTPase activity of RHEB (Ras homolog, mTORC1 binding), while RHEB binding to GTP causes the activation of mTORC1 [86]. Although TSC1 is not a GAP (RAS p21 protein activator 1), it stabilizes TSCs to maintain GTPase activity [82]. Thus, TSC1 or TSC2 ablation is supposed to strengthen RHEB-GTP stability, and consequently, abnormal activation of mTORC1 results in arrested differentiation in the early stage [84,87]. In some situations, mTORC1 hyperactivity causes distinctly delayed myelination and remyelination after injury [87]. Surprisingly, TSC1 deletion is detrimental to OL myelination [88,89].

mTORC1 activity also is downregulated by RAB35 (RAB35, member RAS oncogene family), a Ras-related GTPase controlling myelin growth through MTMR2 (myotubularin-related protein 2) and MTMR13 [90]. Accordingly, disruption of *Rab35* causes hypermyelination via elevation of PI3P signaling and mTORC1 hyperactivation [90].

Some factors can switch the PI3K pathway to the MAPK pathway, such as Integrin α6 in OLs, a receptor for laminins in the neuronal axon. When the two types of molecules contact each other, myelin-forming OLs activate the switch in survival signaling dependence [91], which reverses neuregulin’s inhibition in OL differentiation, subsequently promoting the myelination process [91,92].

### 3.2. ERK1/2

The ERK1/2 advocates myelin wrapping during myelination and remyelination, and maintained OL ERK1/2 activation causes hypermyelination in the CNS [1] (Figure 3). ERK1/2 contributes to remyelination after the demyelination induced by lysophosphatidylcholine (LPC) injection into the corpus callosum [93]. MEK1 (MAPK kinase 1) is the upstream activator of ERK1/2 and displays declined expression in experimental autoimmune encephalomyelitis (EAE) induction [1]. Accordingly, maintained ERK1/2 activation upgrades myelin thickness after the LPC injection [94,95]. MEK inhibitors, such as PD0325901, AZD6244, AZD8330, CI-1040, and U0126 [96,97,98], significantly rectify OPC differentiation in a time- and dose-dependent manner, suggesting that regulation of the MAPK–ERK signaling pathway is sufficient to accelerate OL generation, facilitating myelin sheath formation [96].

### 3.3. GPR37

GPR37 is highly expressed in OLs and significantly intensified during OL differentiation and myelination (Figure 3). GPR37, also known as PAELR (Parkin-associated endothelin B-like receptor), has been identified as a substrate of parkin (an E3 ubiquitin ligase) [99]. Although the OPC number was not affected in *Gpr37* null mice, GPR37 is regarded as an inhibitor of OL differentiation and myelination. Yang HJ et al. found that GPR37 restricts OL differentiation and hypermyelination by suppressing the cAMP-dependent Raf-MAPK-ERK1/2 cascade [35]. *Gpr37* knockout leads to dramatically diminished MAG expression in the mouse brain. The mutant mice exhibit strikingly enlarged myelin loss during the cuprizone demyelination model without impacting the number of OPCs and OLs [100]. GPR37 suppresses activation of the cAMP-dependent Raf-MAPK-ERK1/2 cascade via inhibiting AC (adenylate cyclase) [35].

### 3.4. SOX10-MYRF

MYRF (myelin regulatory factor) is a membrane-bound transcriptional factor on the endoplasmic reticulum (ER). It forms homo-trimers in the ER [101] and then undergoes self-cleavage via the intrinsic peptidase [102,103,104]. TMEM98 (transmembrane protein 98) can block MYRF self-cleavage in vitro and in vivo [105,106]. Following the self-proteolysis, the homo-trimer of MYRF N-terminal fragments is translocated to the nucleus and binds the motif to activate the transcription of myelin genes [102,103,107,108,109,110,111,112].

MYRF is required to initiate and maintain myelination [8,113] (Figure 3). *Myrf* knockout mice sustain the premyelinating stage, leading to myelination failure and postnatal death [10,114], a similar phenotype to that in *Olig1* null mice [115] and *Sox10* mutant Zebrafish [116]. Loss of *Myrf* in OPCs does not alter OPC proliferation and recruitment after demyelination but impairs remyelination because of diminishing OL differentiation [107,117]. In addition, OLs deriving from OPCs with *Myrf* deletion produce few myelin proteins in response to demyelination [107]. Not surprisingly, conditioned knockout *Myrf* in mature OLs leads to a dramatic downregulation of myelin gene expression and impairment of myelin sheaths [113,117].

*Myrf* expression is enormously intensified during the initiation of OL differentiation, and SOX10 acts as a *Myrf* gene enhancer [118]. SOX10 binds the first intron of *Myrf*, which is also the region regulated by OLIG2 [119]. Once induced by SOX10, MYRF redirects SOX10 to myelin gene expression [112]. Therefore, the feedback and forward regulatory loops compose an essential molecular circuit for OL differentiation and maturation. Surprisingly, despite the importance of MYRF in OL differentiation and maturation, most of the genetic mutations of *Myrf* do not cause apparent myelin-related human diseases [120,121,122,123,124].

### 3.5. NRG1

NRG1 (Neuregulin-1) promotes OPC migration, proliferation, and differentiation into OLs [125,126,127], and NRG1/ErbB (Erb-b2 receptor tyrosine kinase) signaling (Figure 3) is required for OL survival and maturation [128,129,130].

NRG1 has more than 30 splice isoforms, sharing an EGF-like function domain to activate the receptors, ErbB2/ErbB3 heterodimer, or ErbB4 homodimer [131,132,133]. Immature NRG1 is a transmembrane protein that releases soluble N-terminal moieties containing the EGF-like domain after proteolytic processing [133]. Soluble NRG1 is a mitogen for OLs, provides an axonal signal for OL survival and increases myelination [134]. Inhibiting its receptors reversed the positive effects of the administration of soluble NRG1 [125]. BACE1 (Beta-site APP-cleaving enzyme 1) cleaves NRG1 type Ⅰ and type Ⅲ. *Bace1* deficiency leads to hypomyelination and impairs remyelination [129].

NRG1, acting through ErbB, is an important regulators in OPC proliferation and OL differentiation during development [135]. NRG1-ErbB modulates myelin-related gene expression depending on the PI3K-AKT-mTOR pathway [132,136,137]. Recently, Ding Z et al. found that NRG1 can convert astrocytes into OL lineage cells via the PI3K-AKT-mTOR signaling activation and eventually improves remyelination [138]. Increasing AKT activity of the OLs in *Bace1* null mice is sufficient to normalize myelination without inducing hypermyelination [139].

*Nrg1 type Ⅲ* null mice died at birth owing to the deficiency of functional neuromuscular junction, which also hampers the analysis of NRG1 in the CNS [140,141]. Although OLs normally differentiate when cocultured with *Nrg1 type Ⅲ* null dorsal root ganglia, the myelination of dorsal root ganglia is impaired in the *Nrg1 type Ⅲ* deficient mice [142]. Intriguingly, heterozygous *Nrg1 type Ⅲ* mutant mice exhibit hypomyelination in the brain, but myelination in the spinal cord and the optic nerve is normal [142]. However, others have not found CNS hypomyelination in heterozygous *Nrg1 type Ⅲ* mutant or *Nrg1* null mice [143]. Surprisingly, mice overexpressing *Nrg1 type Ⅲ* have hypomyelinated axons in the CNS [143]. *Nrg1 type Ⅲ* overexpression in the spinal cord improves motor function and increases motor neuron survival in mice with amyotrophic lateral sclerosis [144].

### 3.6. BDNF

CNS myelinating cells develop from slowly dividing adult progenitor cell OPCs through a premyelinating OL stage before maturing into myelinating OLs. *Bdnf* (brain-derived neurotrophic factor) heterozygous mice display hindered myelination in the CNS [145] (Figure 3). BDNF functions via two different classes of transmembrane receptors: TRKB (tropomyosin-related kinase receptor B) and p75NTR (p75 neurotrophin receptor). Maintaining BDNF enhances myelination, but it cannot be attenuated in the *p75Ntr* knockout mice [145]. Therefore, p75NTR is not necessary for promoting myelination. Conversely, BDNF cannot rescue the impeding myelination induced by the impairment of TrkB signaling [145].

### 3.7. GlcNAc

Recently, Michael et al. [146] found that GlcNAc (N-acetylglucosamine) is essential in triggering OL differentiation. N-glycan branching and GlcNAc support OPC differentiation into OLs via suppressing PDGF-α. Furthermore, primary myelination in newborn pups is strengthened when the lactating mice are supplemented with oral GlcNAc. Conversely, by blocking N-glycan branching, primary myelination is impeded. Intriguingly, oral GlcNAc protects neuronal axons from damage in the cuprizone-induced demyelination mouse model via enhancing myelin reparation [146].

### 3.8. OLIG2

OLIG2 is regarded as a marker of OL family cells, although it is also expressed during development in motoneurons, subgroups of astrocyte precursors, and Purkinje cell precursors [147,148,149,150,151]. OLIG2 acts as a binding upstream enhancer to induce the expression of target genes such as *Nkx2.2* (NK2 homeobox 2) and *Sox10*. Conditional knockout of *Olig2* in embryonic neural stem cells confines OL differentiation without the alteration of OL specification, resulting in hypomyelination [152].

OLIG2 is critical to OPC specification and OL differentiation. When cortical OPCs are conditional knockout *Olig2*, the OPCs are transformed into astrocytes [153]. OPCs are completely absent from most regions of the CNS in *Olig2* knockout mice [154,155,156,157]. However, OPCs are still present in dramatically reduced numbers in the forebrain and hindbrain of *Olig2* knockout mice [154,158]. Conditioned knockout of *Olig2* in OPCs leads to hypomyelination because of the limitation of OL differentiation, while conditional knockout of *Olig2* in immature OLs accelerates OL myelination by facilitating maturation [158].

Though *Olig2* is closely related to *Olig1* [159], *Olig1* provides little compensation for *Olig2* loss [147]. OLIG2 forms a homodimer or a heterodimer to induce OPC specification [148]. Moreover, OLIG2 exhibits versatile functions in OPCs and OLs via post-translational modification, which was reviewed thoroughly by H Li and WD Richardson [151].

### 3.9. PDE

PDEs (phosphodiesterases) have been implicated in OL maturation and myelination in the CNS (Figure 3). Inhibitors of PDEs, such as PDE1 inhibitor vinpocetine [160] and PDE5 inhibitor sildenafil [161], not only spoil inflammation but also exert a negative impact on the CNS OL differentiation processes, including diminishing myelin gene expression and enhancing myelination negative transcriptional regulators (such as ID2 and ID4) [161].

## 4. OL Maturation

OPC proliferation and OL differentiation are the two main stages in the myelination process, and factors commonly have converse roles in these two stages. However, some genes mainly regulate OL maturation, and their mutation induces hypomyelination or hypermyelination without an obvious function in the proliferation and differentiation of OLs.

### 4.1. DDIT4

DDIT4 (DNA damage inducible transcript 4, also known as REDD1/Dig2/RTP801) is a negative regulator of myelination [83]. DDIT4 maximum expression is in line with the peak activity of AKT and DLG1. Moreover, *Ddit4* deficiency provokes hypermyelination by enhancing mTOR activation and enlarged myelin thickness. Intriguingly, *Ddit4*-deficient mice do not present myelin out-folding (depending on PIP3) or macula (depending on AKT) [83].

### 4.2. JAM2

Neuronal JAM2 (junction adhesion molecule 2) is sufficient and necessary in the somatodendritic membrane to inhibit OL myelination in the neuronal cell body [77,162], while Galectin-4 is an inhibitor in the axons [163]. Galectin-4 is specifically sorted into segmental domains along the axon membrane, and OLs do not deposit myelin on Galectin-4 covered surfaces, leading to long unmyelinated axon segments [163]. After the JAM2 extracellular portion is fused to the immunoglobulin Fc region, the formed JAM2-Fc is added into cultured OLs, with higher JAM2-Fc binding to MBP+ myelinating OLs than to OPCs, suggesting that the JAM2 receptor is upregulated on the surface during OL differentiation [77,162]. Furthermore, soluble JAM2-Fc arrests myelin formation in cultured OLs from wild-type mice [77,162]. Intriguingly, recent studies have found that JAM2 is also associated with primary familial brain calcification, an uncommon degenerative neurological disease due to abnormal calcium phosphate deposits in the brain [164,165]. In *Jam2* null mice, the neuronal soma is sheathed, and contactin-associated protein, which typically localizes only with the paranodal structures of the Nodes of Ranvier, clusters on the neuronal somatic member [77].

### 4.3. PKD1

PKD1 (protein kinase D1), a serine/threonine kinase belonging to the calcium/calmodulin-dependent kinase family, is implicated in OL maturation, except for its role in tumor progression [166,167]. *Pkd1* homozygous deficiency is lethal to the mutant mice, and *Pkd1* heterozygous mutant mice exhibit quickly inducible epilepsy and hypermyelination, supporting the finding of the epilepsy patient [168]. In addition, PKD1 can elevate functional synapse formation by enhancing N-cadherin’s stability in an activity-dependent manner [169].

### 4.4. TTR

TTR (Transthyretin) binds and transfers thyroid hormones in cerebrospinal fluid and blood. *Ttr* mutation is related to familial amyloid polyneuropathy, a neurodegenerative disorder with TTR deposition in the peripheral nervous system [170,171]. However, *Ttr* null mice exhibit hypermyelination, magnified OL density in the corpus callosum, and anterior commissure during postnatal development [172]. Moreover, *Ttr* deficiency magnifies OPC migration and proliferation with debased apoptosis [172]. Intriguingly, TTR is expressed in OPCs, and boosting the pAKT level in OLs may be the mechanism of hypermyelination [172]. During remyelination in the adult mouse corpus callosum, *Ttr* null mice exhibit an expedited remyelination rate, preferentially remyelinating small axons [173]. Moreover, *Ttr* null mice display thicker myelin than wild-type mice [173].

### 4.5. LINGO-1

LINGO-1 (leucine-rich repeat and Ig-like domain-containing Nogo receptor interacting protein 1), a transmembrane protein, is expressed explicitly in OLs and neurons, serving as a potent negative modulation of axonal myelination and regeneration in the CNS [6,174]. Downregulating LINGO-1 functions [6], such as *Lingo-1* RNAi [175], sh-RNA [174,176], anti-LINGO-1 antibody [177,178,179,180,181], dominant-negative LINGO-1, or soluble LINGO-1-Fc [182,183], improves OL differentiation and myelination, accompanied by prolonged process length and augment branching, and downregulated RhoA [181,184] activity is the potential mechanism [6]. Conversely, *Lingo-1* overexpression results in RhoA activation, negatively regulating OL differentiation and myelination [6]. Neuronal LINGO-1 is a critical component of the Nogo receptor complex, restricting axonal growth via RhoA. The Nogo receptor is absent in OLs, and consequently, LINGO-1 prevents OL myelination through intercellular interactions with self-association in the trans [183] or cytoplasmic gelsolin signaling pathway [179].

### 4.6. N-WASP

N-WASP (Neural Wiskott–Aldrich syndrome protein) is essential for myelin wrapping in Schwan cell and OL myelination [185,186,187,188]. Conditional knockout *N-Wasp* continues to ensheathe the onset myelinating axons but fails to extend circumferentially to elaborate myelin, and the affected mice demonstrate apparent motor deficits without progress [187]. In *N-Wasp*-deficient nerves, most cells arrest at the premyelinating stage and subsequently fail to myelinate, with occasional misfolding myelin forming unusually short internodes and thin myelin sheaths [186]. Strikingly, *N-Wasp* deficiency leads to hypomyelination and induces remarkably focal hypermyelination, representing long myelin out-folds enclosing neuronal cell bodies and unmyelinated axons [185].

### 4.7. PRMT5

PRMT5 (protein arginine methyltransferase 5) is a histone arginine methyltransferase catalyzing histone H4R3 methylation. Although suppression or knockout of *Prmt5* does not affect OPC proliferation, it attenuates OPC survival and differentiation leading to hypomyelination [189]. Its potential mechanism is heightened nuclear acetylation of H4K5 following histone H4R3 methylation reduction, which can be rescued via bridling histone acetyltransferases [189].

### 4.8. ZEB2

ZEB2 (zinc finger E-box binding homeobox 2, also known as Zfhx1b and Sip1), a transcription factor, contributes to many essential neurodevelopmental processes [190,191,192,193]. ZEB2 heterozygous mutation in humans leads to Mowat–Wilson syndrome [190,193]. *Zeb2* knockout mice have severe impairment of myelination, failing to express myelin genes, and ZEB2 may be one of the direct transcriptional targets of *Olig2* [194,195]. Moreover, ZEB2 controls the onset of Schwann cell differentiation by recruiting HDAC1/2 and nucleosome remodeling and deacetylase complex co-repressor complexes in mice [192].

### 4.9. PAD2

Citrullination, a modification converting peptidyl-arginine residues to peptidyl-citrulline, has been associated with the etiology of several diseases, including inflammation in the CNS [196]. Citrullination by PAD2 (peptidyl-arginine deiminase 2) [197] contributes to OL differentiation and myelination by modifying myelin and chromatin-related proteins [198]. In *Pad2* transgenic mice, homozygous mice exhibit thinner myelin and more severe focal demyelination than heterozygous mice [199]. Overexpression of *Pad2* increases levels of TNF-α (tumor necrosis factor-α), TNF-α induces predominantly cytosolic PAD4 translocation into the nucleus, and high citrullination of histones by PAD4 causes irreversible changes to OLs, which may contribute to apoptosis [199,200]. Moreover, citrullination of MBP by PAD2 leads to reduced interaction of the arginine residues with negatively charged lipids, forming incompact sheaths lacking an attraction between the MBP and lipids, and consequently, the myelin becomes unstable or remains immature [201].

### 4.10. NPC1

CNS hypomyelination is one of the pathological characteristics of Niemann–Pick Type C disease (NPC), a rare childhood-onset neurodegenerative disorder due to mutations of *NPC1* (NPC intracellular cholesterol transporter 1) or *NPC2* [202]. *Npc1-*deficient mice display hypomyelination and delayed myelination caused by hampered OL maturation [202,203]. NPC patients suffer abnormally swollen axons and intracellular lipid accumulation [204]. A deficiency of NPC1, a transmembrane protein essential for mobilizing cholesterol from late endosomes and lysosomes, in neurons alone does not affect the density of OPCs but results in an arrest of OL maturation [204]. Deletion of *Npc1* in OLs leads to a delay rather than a block of myelination [204]. *Npc1* is also required for CNS myelin maintenance because OL-conditioned knockout *Npc1* in aged mice causes late-stage myelin loss, followed by secondary Purkinje neuron degeneration [204].

## 5. Conclusions

The generation of myelin by OLs is crucial to the central nervous system of vertebrates. OPCs originally arise from neuroepithelial precursor cells and then proliferate and differentiate into premyelinating OLs, subsequently differentiating into myelinating OLs. OPC proliferation, OL differentiation, and maturation are the critical processes of OL myelination in the CNS. The extracellular signals, intracellular signaling pathways, and transcription factors regulating myelination are increasingly well-established, which shed light on human demyelinating diseases. The regulation of myelination is a very delicate process. Too much or too little, too early or too late—both extremes affect the function of the entire organism. The myelination processes closely connect with each other, and factors involved in myelination or remyelination may execute multiple functions, even exhibiting contradicting roles in a similar process, depending on the research methods. In order to give a clear description, the factors discussed here are summarized in Table 1.

The next challenge is identifying the common critical intracellular and transcriptional factors for OPC proliferation, OL differentiation, and maturation pathways that can be targeted. The precise regulation of related signaling pathways will be challenging, and associated treatment plans need to be considered. Achieving a thorough understanding of the factors regulating CNS myelination will improve our knowledge about human demyelinating diseases, and the heightened understanding will enable more effective treatments.

## Figures and Tables

**Table 1:** Functions of the factors in the specific stages of OL development.

Factors	Potential Pathway	OPC Proliferation	OL Differentiation	OL Maturation	References
HDAC	HDAC-TCF7L2		+		[16,19,22]
WNT	WNT-β-Catenin-TCF7L2	+	−		[17,18,19]
GPR17	GPR17-ID2/ID4; GPR17-OLIG2; GPR17-ERK1/2	+/−	−	−	[13,42,43,50,51,52,54,55,56]
GPR56	GPR56/Gα12/13-RhoA	+	−	−	[33,34,36,37,38,39]
LINGO-1	LINGO-1-RhoA		−	−	[6,174,175,176,177,178,179,180,181,182,183]
TG2	TG2-Laminin-GPR56	+			[40]
ERK1/2	MEK-ERK1/2		+	+	[93,94,95,96,97,98]
GPR37	GPR37-cAMP-Raf-MAPK-ERK1/2		−	+/−	[35,100]
OLIG2	OLIG2-NKX2.2; OLIG2-SOX8/10	+	+	+/−	[152,158]
ZEB2	OLIG2-ZEB2			+	[194,195]
SOX10-MYRF	SOX10-MYRF		+	+	[102,103,107,109,110,111,112,113,114,117,118]
AKT-mTOR	PI3K-AKT-mTOR		+	+	[78,79,80,81,90]
DDIT4	DDIT4-mTOR			−	[83]
NRG1	NRG1/ErbB- PI3K-AKT-mTOR	+	+	+/−	[125,126,127,128,129,130,134,135,136,137,138,142,143]
TTR	TTR-pAKT			−	[172,173]
BDNF	BDNF/TrkB		+	+	[145]
FGF2	FGF2/FGFR1	+	−	+/−	[23,24,25,28,29,31]
GlcNAc	GlcNAc-PDGF-α		+	+	[146]
JAM2	\			−	[77,162]
NG2	\	+	−		[9,10,11]
NOTCH1	NOTCH1-HES5	−	−	−	[63,64,65,66,67]
NPC1	\			+	[202,203,204]
N-WASP	\			+	[185,186,187]
PAD2	PAD2-TNF-α; PAD2-MBP		−	−	[196,198,199,200,201]
PDE	\		+	+	[160,161]
PKD1	\			−	[168]
PRMT5	\		+	+	[189]
SOX2	\	+	+/−		[59,60,61,62]
SHH	SHH-BOC	+		+	[69,70,71,72,73]

(Notes: Pathways sharing one or more of the same factors are shown with the same background color. +, activation; −, inhibition; +/−, activation or inhibition, depending on the different research studies; \, unclear).

## Data Availability

Not applicable.

## Abbreviations

5-FU	5-Fluorouracil
AC	Adenylate cyclase
ADGRG1	Adhesion G protein-coupled receptor G1
AKT	AKT serine/threonine kinase 1
BACE1	Beta-site APP-cleaving enzyme 1
*Bdnf*	Brain-derived neurotrophic factor
BFPP	Bilateral frontoparietal polymicrogyria
BMP	Bone morphogenetic protein
BOC	BOC cell adhesion associated, oncogene regulated
BPA	Bisphenol-A
cAMP	Cyclic adenosine monophosphate
CNP	2,3-cyclic nucleotide 3-phosphodiesterase
CNS	Central nervous system
CREB	cAMP responsive element binding protein 1
DDIT4	DNA damage inducible transcript 4
DLG1	Discs large MAGUK scaffold protein 1
ER	Endoplasmic reticulum
*ErbB*	Erb-b2 receptor tyrosine kinase
FGF2	Fibroblast growth factor 2
GAP	RAS p21 protein activator 1
GlcNAc	N-acetylglucosamine
*Gli1*	GLI family zinc finger 1
GPR56	G protein-coupled receptor 56
HDAC	Histone deacetylase
HES5	Hes family bHLH transcription factor 5
ID2	Inhibitor of DNA binding 2
JAM2	Junction adhesion molecule 2
LEF	Lymphoid enhancer binding factor
LINGO-1	Leucine-rich repeat and Ig-like domain-containing Nogo receptor interacting protein 1
LPC	Lysophosphatidylcholine
MAG	Myelin-associated glycoprotein
MAPK	Mitogen-activated protein kinase
MBP	Myelin basic protein
MEK1	MAPK kinase 1
*Mib1*	MIB E3 ubiquitin protein ligase 1
MTMR2	Myotubularin-related protein 2
mTOR	Mechanistic target of rapamycin
MYRF	Myelin regulatory factor
NG2	Chondroitin sulfate proteoglycan neuron-glia antigen 2
*Nkx2.2*	NK2 homeobox 2
NOTCH1	Notch receptor 1
NPC	Niemann–Pick Type C disease
NPC1	NPC intracellular cholesterol transporter 1
NRG1	Neuregulin-1
N-WASP	Neural Wiskott–Aldrich syndrome protein
p75NTR	p75 neurotrophin receptor
OL	Oligodendrocyte
OLIG2	Oligodendrocyte transcription factor 2
OPC	Oligodendrocyte progenitor cell
PAD2	Peptidyl-arginine deiminase 2
PAELR	Parkin-associated endothelin B-like receptor
PDEs	Phosphodiesterases
PDGFRα	Platelet-derived growth factor receptor α-subunit
PI3K	Phosphatidylinositol-4,5-bisphosphate 3-kinase
PKA	Protein kinase A
PKD1	Protein kinase D1
PPH	Primary palmar hyperhidrosis
PTEN	Phosphatase and tensin homolog
RAB35	RAB35, member RAS oncogene family
RAPTOR	Regulatory-associated protein of MTOR complex 1
RHEB	Ras homolog, mTORC1 binding
RhoA	Ras homolog family member A
RICTOR	RPTOR independent companion of MTOR complex 2
SHH	Sonic hedgehog
*Smo*	Smoothened, frizzled class receptor
SOX2	SRY (sex-determining region Y)-box transcription factor 2
TCF7L2	Transcription factor 7-like 2
TG2	Transglutaminase-2
TMEM98	Transmembrane protein 98
TNF-α	Tumor necrosis factor-α
TRKB	Tropomyosin-related kinase receptor B
TSC1	Tuberous sclerosis protein 1
TTR	Transthyretin
WNT	Wingless-type MMTV integration site family
*Xaf1*	XIAP-associated factor 1
ZEB2	Zinc finger E-box binding homeobox 2
